# Blood Extracellular Vesicles Beyond Circulating Tumour Cells: A Valuable Risk Stratification Biomarker in High-Risk Non-Muscle-Invasive Bladder Cancer Patients

**DOI:** 10.3390/biomedicines12102359

**Published:** 2024-10-16

**Authors:** Valentina Magri, Luca Marino, Francesco Del Giudice, Michela De Meo, Marco Siringo, Ettore De Berardinis, Orietta Gandini, Daniele Santini, Chiara Nicolazzo, Paola Gazzaniga

**Affiliations:** 1 Department of Pathology, Oncology and Radiology, Policlinico Umberto I Hospital, “Sapienza” University of Rome, 00161 Rome, Italy; 2 Department of Mechanical and Aerospace Engineering, Sapienza University of Rome, 00184 Rome, Italy; luca.marino@uniroma1.it; 3 Emergency Medicine Unit, Department of Emergency-Acceptance, Critical Areas and Trauma, Policlinico “Umberto I”, 00183 Rome, Italy; 4 Department of Maternal Infant and Urologic Sciences, “Sapienza” University of Rome, Policlinico Umberto I Hospital, 00161 Rome, Italy; francesco.delgiudice@uniroma1.it (F.D.G.); ettore.deberardinis@uniroma1.it (E.D.B.); 5 Department of Molecular Medicine, “Sapienza” University of Rome, 00161 Rome, Italy; michela.demeo@uniroma1.it (M.D.M.); marco.siringo@uniroma1.it (M.S.); orietta.gandini@uniroma1.it (O.G.); chiara.nicolazzo@uniroma1.it (C.N.); paola.gazzaniga@uniroma1.it (P.G.); 6 Department of Medical-Surgical Sciences and Biotechnologies, “Sapienza” University, 04100 Latina, Italy; daniele.santini@uniroma1.it

**Keywords:** extracellular vesicle, circulating tumour cell, non-muscle invasive bladder cancer, Cellsearch^®^ system, ACCEPT

## Abstract

Non-muscle-invasive bladder cancer (NMIBC) prognosis varies significantly due to the biological and clinical heterogeneity. High-risk stage T1-G3, comprising 15–20% of NMIBCs, involves the lamina propria and is associated with higher rates of recurrence, progression, and cancer-specific mortality. In the present study, we have evaluated the enumeration of tumour-derived extracellular vesicles (tdEVs) and circulating tumour cells (CTCs) in high-risk NMIBC patients and their correlation with survival outcomes such as time to progression (TTP), and cancer-specific survival (CSS). Eighty-three high-risk T1-G3 NMIBC patients treated between September 2010 and January 2013 were included. Blood samples were collected before a transurethral resection of the bladder (TURB) and analysed using the CellSearch^®^ system. The presence of at least one CTC was associated with a shorter TTP and CSS. Extending follow-up to 120 months and incorporating automated tdEV evaluation using ACCEPT software demonstrated that tdEV count may additionally stratify patient risk. Combining tdEVs and CTCs improves risk stratification for NMIBC progression, suggesting that tdEVs could be valuable biomarkers for prognosis and disease monitoring. Further research is needed to confirm these findings and establish the clinical significance of tdEVs in early-stage cancers.

## 1. Introduction

Non-muscle-invasive bladder cancers (NMIBCs) account for approximately 75–80% of all cases of bladder cancer [1]. Patients with NMIBCs exhibit a wide range of clinical outcomes due to the considerable biological and clinical heterogeneity [2]. The high-grade stage T1 group accounts for about 20% of cases and it is characterized by invasion into the lamina propria whose depth and extent carries significant prognostic importance associated with increased rates of recurrence, progression, and cancer-specific mortality [3,4].

The 2021 European Association of Urology (EAU) scoring model allows the assessment of individual patients’ risk of progression by stratifying them into risk groups. Based on their calculated risk, patients are categorized into low, intermediate, high, or very high-risk groups, which is critical for determining upfront radical interventions or the type and schedule of further adjuvant intravesical treatment [5].

The accurate prediction of the risk of progression is mandatory in NMIBCs management to avoid undertreatment or overtreatment. The identification of robust prognostic biomarkers could significantly improve therapeutic decision-making and, although advances in molecular markers have enhanced our understanding of bladder cancer biology, their clinical utility remains restricted. To date, relevant studies are ongoing to identify circulating biomarkers to monitor subclinical systemic disease and identify therapeutic targets [6,7,8,9,10]. In particular, circulating tumour cells (CTCs), have been previously recognized as surrogate markers for early disease dissemination and promising prognostic biomarkers in different type of solid malignancies, including non-muscle-invasive bladder cancer [11,12,13].

In a previous study performed in patients with high-grade T1 NMIBCs, the presence of at least one CTC was found to be independently associated with clinical outcome, attesting to the positive predictive value of CTCs for disease recurrence and progression, as well as for overall survival with a median follow-up time of 63 months [13].

Recently, alongside CTCs, a growing body of evidence has demonstrated the particular relevance of additional circulating entities such as tumour-derived extracellular vesicles (tdEVs) [14]. These elements can provide valuable insights into tumour biology, progression, and treatment response. Despite the fact that extracellular vesicles (EVs) were originally considered as simple products of cellular degradation and cell death, recently their role as mediators of intercellular communication has been fully recognized [15].

This paradigm change is related to the increased comprehension of the fundamental role of EVs, including tdEVs, in transferring a wide range of biologically active molecules, including nucleic acids, proteins, and lipids, between cells in targeted and regulated processes [16].

The activity of tdEVs, as biologically active carriers, makes them potentially useful biomarkers for early diagnosis, prognosis, and disease monitoring. Moreover, the possibility of isolating, enumerating and analysing tdEVs from non-invasive biological samples, such as blood or urine, makes them attractive for clinical applications [17,18].

The comprehensive analysis of Nanou et al. [19] conducted on the tdEVs in castration-resistant prostate cancer (CRPC), metastatic breast cancer (MBC), and metastatic colorectal cancers (mCRC), has highlighted their prognostic potential equivalent to that of CTCs. Furthermore, tdEV count was found to be at least one order of magnitude higher compared to that of CTCs. The higher number of tdEVs can provide additional insights into tumour progression, metastasis development, risk stratification, and treatment response, thus improving the ability to predict patient outcomes and better tailor personalized therapeutic strategies [20]. While recent studies predominantly focused on the enumeration and analysis of tdEVs in locally advanced or metastatic settings, the prognostic role of tdEVs in early-stage cancers remains largely unexplored.

Currently, CellSearch^®^ is the only FDA-approved platform for CTC detection in blood samples from patients with castration-resistant prostate cancer (CRPC), metastatic breast cancer (MBC), and metastatic colorectal cancer (mCRC). CTC enumeration using CellSearch^®^ can be performed manually or automatically. The manual approach is time-consuming depending on the operator’s interpretation and experience, often leading to potential variability and uncertainty in results [21]. New approaches for the automatic detection and counting of CTCs are currently available, reducing potential bias due to operator variability. Specifically, the enumeration of tdEVs based on the characteristics of CellSearch^®^-generated thumbnails can only be accurately achieved through a software-based procedure (ACCEPT: Automated CTC Classification, Enumeration, and PhenoTyping) [22], an image analysis tool that includes characterizing markers on pre-scored CTCs to achieve a comprehensive detection of all events [23].

The present study aimed to evaluate the prognostic significance of tdEVs on key survival endpoints including time to first/second recurrence (TFR and TSR), progression (TTP), and cancer-specific survival (CSS) in NMIBC. Furthermore, the prognostic significance of tdEV combined with CTC enumeration was further explored. An additional outcome is the extension of the follow-up of a previous study to improve the accuracy of long-term survival rate assessments.

## 2. Materials and Methods

### 2.1. Methodology Details

The present study was performed on 83 patients enrolled in a single-centre analysis, with pathologically confirmed high-risk T1-G3 NMIBC (histological grading was based on the 2004 WHO classification), treated between September 2010 and January 2013, according to standard international guidelines (transurethral resection of the bladder (TURB), re-TURB, and adjuvant intravesical Bacillus Calmette-Guérin (BCG) induction plus maintenance for 1–3 years). The follow-up was obtained through a cystoscopy and urinary cytology every 3 months and a computed tomography with contrast medium every 12 months.

The circulating elements (i.e., CTCs and tdEVs) were isolated before the first TURB from 7.5 mL of blood collected into evacuated blood draw tubes through the CellSearch^®^ system (Menarini Silicon Biosystems, Castel Maggiore, Bologna, Italy), according to the manufacturer’s guidelines (ref). The CellSearch^®^ system employs the CellSearch^®^ Epithelial Cell Kit, enabling CTC enrichment via an anti-EpCAM-antibody-coated ferrofluid reagent, followed by staining with cytokeratins (CKs), 4′-6-Diamidino-2-phenylindole (DAPI), and CD45. Peripheral blood was collected in a CellSave tube with EDTA and a fixative, and processed within 72 h. CellSearch^®^ utilizes immunomagnetic separation and fluorescent labelling techniques to identify and enumerate CTCs and allows the acquisition of thumbnails containing signals for CK-PE (cytokeratin-phycoerythrin, a protein marker commonly found in epithelial cells, including cancer cells) and DAPI (4′,6-diamidino-2-phenylindole, a fluorescent dye that binds to DNA, facilitating the visualization of cell nuclei). CD45 serves as a marker to differentiate white blood cells from circulating tumour cells (CTCs). By combining all of these markers, CellSearch^®^ effectively detects and enables the operator to enumerate CTCs. However, CellSearch^®^ does not generate thumbnails with positive objects solely for CK-PE; consequently, it does not allow for the enumeration of tdEVs [19]. This challenge can be overcome by using software like ACCEPT, an open-source software image analysis algorithm designed to analyse recorded images of the CellSearch^®^ platform, which is capable of identifying all fluorescent images and analysing morphology and fluorescence intensity in each CellSearch^®^ channel. By employing advanced image analysis algorithms and machine learning techniques, ACCEPT and similar software can provide more accurate and reproducible results. As a result, ACCEPT software represents a valuable resource for researchers and clinicians, facilitating the use of CTCs and tdEVs as biomarkers in research and clinical settings. The tdEV counts were integrated with the CTC values previously obtained [12].

The present study extends the previous analysis, limited to CTCs, to the automatic evaluation of tdEVs obtained with ACCEPT, [22]. Initially aimed at CTC evaluation, the application of the software has been further extended to enumerate tdEV circulating elements, adopting the following set of parameters: a size between one and twelve micro-m, the expression of epithelial cell features (EpCAM and cytokeratins), a lack of leucocyte-specific marker (CD45), and nuclear staining. The procedure adopted to identify the software parameters has been recently described in different disease settings [24]. Currently, the set of ACCEPT gate parameters can be considered well-tested and available in the literature [22].

### 2.2. Statistical Analysis

A statistical analysis was adopted to evaluate the survival parameters, related to clinical information such as TTP, TFR, TSR, and CSS, following the Kaplan–Meier product-limit method, with the log-rank test used to assess differences between subgroups. Significance was defined at the *p* ≤ 0.05 level. Statistical analysis was performed with SPSS software (version 24.0; SPSS Inc., Chicago, IL, USA).

## 3. Results

The baseline characteristics (mean age, gender distribution, and smoking habit) of the 83 patients enrolled in this study are presented in Table 1.

In the whole population enrolled for this study, we identified CTCs (cut-off ≥ 1) [13] in 15 patients (18%); while in the remaining 68 (82%) no CTCs were detected.

The impact of clinical-pathological features on the CTCs and tdEVs counts is reported in Table 2 for the presence of carcinoma in situ (CIS), the tumour size, and the tumour multifocality.

According to CTC enumeration, TTP and CSS were found to be significantly shorter in the group with CTCs ≥ 1 compared to the group with CTCs = 0 (Table 3). The analysis of censored cases vs. events confirmed, for both TTP and CSS, that few patients in the group of CTCs = 0 underwent progression or death.

Notably, all of the 83 patients had CSS = 131.7 ± 7.7 months with 68/83 censured cases, which is quite similar to the CSS value of the CTCs = 0 group of patients (CSS = 148.5 ± 6.6 months).

In Table 4, the distribution of tdEV count is shown. In 74 patients (%), at least one tdEV was detected, while nine subjects (%) had zero tdEVs. Since the proper tdEV prognostic cut-off has never been investigated in the literature, we evaluated different threshold values.

Table 5 and Table 6 show the impact of different thresholds for evaluating the effect of tdEV counts on the mean TTP and CSS values. For each value of the onset count, the differences in TTP and CSS are reported according to the CTCs = 0 or ≥1 value.

The prognostic impact of tdEVs, in addition to CTCs, on the main clinical parameters (TTP, TFR, TSR, and CSS) is reported in Table 5, Table 6, Table 7 and Table 8.

The group CTCs = 0 was further stratified according to tdEV count, with significantly shorter TTP values reported for patients with a positive tdEV count. Remarkably, when CTCs ≥ 1, the TTP was better stratified by including tdEV counts, with significantly shorter TTP values in the group of patients with CTCs ≥ 1 and tdEVs ≥ 7. Notably all patients with CTCs ≥ 1 and tdEVs ≥ 7 had a progression of disease.

Similar results were obtained for the analysis of CSS (Table 6), even if in this case patients with CTCs = 0 had a better prognosis, regardless of tdEV number.

The group of patients with tdEVs ≥ 7 is particularly interesting, as significant differences were observed between patients with CTCs = 0 and CTCs ≥ 1. Notably, with increases in the cut-off value for positive tdEVs (range 7–12), the changes in the value assumed by CSS were almost negligible.

A better stratification with tdEVs was also obtained for TFR and TSR, even if in these cases the results were not found to be statistically significant, as shown in Table 7 and Table 8.

**Figure 1:**
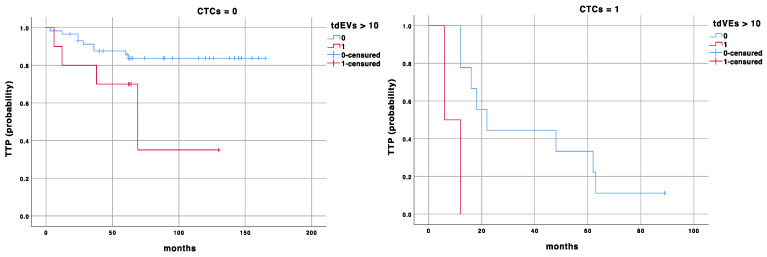
Kaplan–Meier survival curve for time to transition (TTP) according to CTC and tdEV counts.

**Figure 2:**
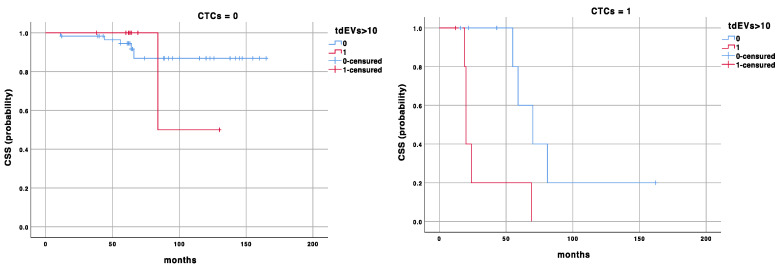
Kaplan–Meier survival curve for cancer-specific survival (CSS) according to CTC and tdEV counts.

In Figure 1 and Figure 2, the Kaplan survival curves for the TTP and CSS probabilities are presented for CTCs = 0 and ≥1. In both cases, the effect of the prognostic cut-off for tdEVs = 10 on the TTP and CSS probabilities is shown.

## 4. Discussion

Approximately 80% of patients diagnosed with bladder cancer have NMIBC, which typically carries a favourable prognosis, with a 10-year CSS rate of 70–85% for high-grade lesions and exceeding 85% for low-grade ones [5]. However, while in low-grade tumours recurrence and progression rates can be as high as 50% and 6%, respectively, the risk of progression to muscle invasive or systemic disease in high-grade tumours can reach up to 45% [24].

To anticipate the short- and long-term risks of disease recurrence and progression, the European Organization for Research and Treatment of Cancer (EORTC) Genito-Urinary Cancer Group (GUCG) devised a scoring system based on clinical and pathological factors. Based on this system, it is possible to further stratify patients and identify those who are at the highest risk of disease progression. However, despite advancements in research and clinical practice, little improvement in the 5-year survival rate for NMIBC has been obtained and frequent recurrences still place a significant burden on public health systems [25,26].

In patients with high-grade NMIBC, the preferred treatment option is intravesical therapy with BCG. Currently, this treatment stands as the most effective due to its ability to lower the risk of recurrence and progression. Unfortunately, a subset of patients do not respond. For these individuals, predictive biomarkers would be of high impact [5]. In this context, liquid biopsy has shown sensitivity in accurately predicting and detecting relapse [13].

In the study, the enrolled patient sample shows demographic data consistent with the scientific literature. This is further supported by data from the GLOBOCAN database of the International Agency for Research on Cancer [27], which indicates that the incidence is approximately four times higher in men than in women, with tobacco smoking as the principal risk factor and a median age at diagnosis of around 70 years. Additionally, the presence of CTCs and tdEVs is correlated with clinicopathological characteristics such as the presence of CIS, tumour size, multifocality, and lymphovascular invasion. These features are well known key predictors of disease recurrence and, consequently, more aggressive disease. Indeed, most risk prediction models incorporate factors such as tumour status (primary or recurrent), the number of lesions, stage, grade, and the presence of carcinoma in situ (CIS) to predict disease recurrence and progression [27,28].

The present study, other than confirming with a longer follow up time (120 months) the relevant prognostic impact of CTCs [12], further proves that the inclusion of tdEVs as liquid biopsy analytes might significantly improve the capability of risk stratification. Moreover, a substantially higher number of tdEVs was found compared to CTCs. The difference is approximatively of one order of magnitude which suggests that adopting tdEVs as a more abundant biomarker might better refine cancer detection or monitoring.

To our knowledge, we, for the first time, demonstrated the clinical usefulness of tdEV enumeration in an early cancer setting, and its significant impact when combined with CTC count. We further settled a specific prognostic cut-off value for tdEVs and demonstrated that in patients with CTC = 0, the stratification with tdEVs ≥ 7 vs. <7 identifies a subgroup of patients with worse TTP (86 vs. 147 months, *p* = 0.01), while in patients with CTC ≥ 1, the comparison of tdEVs ≥ 7 vs. <7 discriminates the subgroup with worse outcomes (TTP: 35 vs. 9 months, *p* = 0.001, and CSS: 85 vs. 30 months, *p* = 0.01); see Figure 1 and Figure 2. These results highlight the utility of a combined analysis of CTCs and tdEVs as prognostic biomarkers capable of improving NMIBC patients’ risk stratification, even in those with a favourable CTC count (CTC = 0).

The importance of tdEV count using a threshold of ≥7 is supported by previously published data. In fact, this cut-off value represents the mean number of tdEVs corresponding to a significant difference between patients and healthy subjects, as reported by Nanou et al. [19,24] for different advanced cancers, such as CRPC, mCRC, and MBC. Notably, the presence of EVs in non-cancer patients has been attributed to liver inflammation or fibrosis. These pathological conditions could lead to an increased secretion of epithelial EVs, as suggested in patients affected by cirrhosis [29]. According to Nanou et al. (2018), tdEVs and EVs were isolated from 129 CRPC patients and 16 healthy subjects, respectively. The mean number of EVs in healthy donors was eight, while the number of tdEVs significantly increased in CRPC patients, with a mean value of 116 (range 1–4920). Similar findings were reported in another study by Nanou et al. [19] on 190 CRPC, 179 MBC, 450 mCRC, and 137 lung cancer patients, compared to 93 healthy subjects. In these studies, an increased tdEV number in the bloodstream was significantly associated with poorer OS across all metastatic cancer types examined. Additionally, the relative numbers of tdEVs were significantly correlated to CTC values in CRPC, mCRC, and MBC, suggesting a potential relationship between these two circulating biomarkers in predicting cancer progression or dissemination. This study highlights the ability to simultaneously isolate and detect CTCs and large tdEVs (1–14 μm) using a single CellSearch assay. TdEVs provide similar prognostic information as CTCs in CRPC, MBC, mCRC, and NSCLC and can further distinguish patients with low or favourable CTC counts. The higher prevalence of tdEVs compared to CTCs may offer a better reflection of tumour diversity and their stability in the bloodstream makes them a valuable biomarker for evaluating tumour characteristics and predicting treatment responses.

On the other hand, in a study performed on 52 breast cancer patients and 22 healthy controls, a high correlation was found between EVs and BMI, but not with any other clinical parameter, suggesting a potential reflection of the “disease state” influenced by both tumour cells and tumour microenvironment. Confidence in identifying only tdEVs could be improved by incorporating more specific antibodies into the staining mixture. This approach would enhance the discrimination between tdEVs and EVs secreted by non-tumour epithelial cells in the bloodstream.

CellSearch^®^ does not generate thumbnails for all images containing only positive objects labelled with CK-PE because the system is not configured to exclusively isolate and display these specific fluorescent signals. Additionally, CellSearch^®^ operates with a limited volume of plasma, which further restricts its ability to accurately capture and count these vesicles. Consequently, it allows the isolation of a limited number of vesicles and cannot achieve precise counting. This limitation affects the accurate quantification of tdEVs [22]. To overcome these challenges, software solutions such as ACCEPT can be utilized. ACCEPT is capable of detecting all fluorescent images and analysing morphology and fluorescence intensity across each of CellSearch^®^’s channels.

An interesting finding, evaluated for the first time in the literature, is the significantly higher count of CTCs and tdEVs in patients with carcinoma in situ (CIS) compared to those without it. This suggests that CIS may be linked to an increased release of these biomarkers into the bloodstream, potentially indicating more aggressive tumour behaviour or an early phase of systemic dissemination. This aspect is further supported by the fact that, without any treatment, approximately 54% of patients with CIS experience progression to muscle-invasive disease. The statistical significance reinforces the importance of CTCs and tdEVs in distinguishing patient groups, offering promising opportunities for early diagnosis and targeted treatment strategies. Additionally, tumour size, multifocality, and lymphovascular invasion are correlated with the presence of CTCs and tdEVs, although this correlation does not reach statistical significance. Recurrence and progression are predicted based on these variables.

The present study has some limitations. The sample size is limited to 83 patients and dates back to tumours diagnosed in 2010–2013 to extend the follow-up and address the lack of markers to differentiate tdEVs from other EVs. In the future, a prospective study with a larger sample size, a proper number of healthy donors and the utilization of the CellSearch^®^’s fourth channel to incorporate a specific marker to discriminate the origin of the vesicles is undoubtedly envisaged.

Moreover, only patients who were strictly G3, according to the old 2004 WHO classification, were enrolled for the study. Some patients that nowadays should be considered high grade could have been skipped. A re-examination was not considered for this study; in fact, the blood samples were acquired only before the first TURB.

Notably, serial samples obtained during the follow-up are advisable in future studies. The evolving understanding of CTCs in the context of bladder tumours is significant, as evidenced by various studies [10,30,31]. Initially, the role of CTCs in predicting outcomes after a transurethral resection of the bladder tumour (TURBT) was minimal. However, recent findings suggest a much greater impact on patient survival. Several factors may explain this shift in perspective. First of all, different studies often involve varying patient demographics, stages of bladder cancer, and treatment histories. Recent improvements in the selection of patient cohorts have led to more homogeneous groups, allowing for clearer insights into the prognostic role of CTCs.

The timing of CTC detection, both pre- and post-TURBT, is also crucial. Early detection prior to TURBT may correlate with worse outcomes due to its association with tumour aggressiveness. In contrast, later detection post-TURBT may indicate microscopic residual disease and potential tumour recurrence. Additionally, CTCs detected after TURBT could arise from an excessive resection of deeper layers or from high infusion fluid pressure during the procedure, necessitating careful attention during the procedure. Furthermore, if CTCs are found post-TURBT, a close monitoring protocol should be established for the patient [31]. Moreover, the role of BCG therapy is also significant. CTCs identified following BCG treatment may indicate resistance to therapy, which is critical for managing bladder cancer. Understanding the relationship between CTC dynamics and BCG efficacy could provide valuable insights into survival outcomes.

In summary, ongoing research into these factors will enhance our understanding of the implications of CTCs and other circulating elements, such as tdEVs, in the management of bladder cancer and their potential as biomarkers for patient prognosis. The presence and count of CTCs and tdEVs could represent new elements to be included in risk stratification models and nomograms to improve the estimation of disease progression and recurrence risk. Future research should include post-TURBT and post-BCG sampling to assess minimal residual disease and possible BCG resistance. This approach could yield valuable insights for optimizing patient management and improving clinical outcomes. Although the exploration of tdEVs is still in its infancy, these circulating elements could be of great impact as a source of information on tumour biology, with a possible significant impact in clinical practice.

## Figures and Tables

**Table 1:** Baseline characteristics of the study sample (N = 83).

**Age (years)**	74 ± 8.58
**Gender (M/F)**	79/4
**Smoking habit (%)**	78

**Table 2:** CTC and tdEV counts for the different clinico-pathological characteristics: CIS, tumour size, multifocal tumour, and lymphovascular invasion.

Clinico-Pathological Characteristics (Numbers of Patients)	CTCs	*p*-Value	tdEVs	*p*-Value
**No-CIS (N = 65)**	2.1 ± 2.9	0.03	5.3 ± 6.4	0.04
**CIS (N = 18)**	21.3 ± 77.4		154.7 ± 573	
**Tumour size < 1 cm (N = 22)**	1.7 ± 2.2	0.16	4.7 ± 3.7	0.14
**Tumour size > 1 cm and <3 cm (N = 42)**	2.3 ± 3.3		7.0 ± 11.5	
**Tumour size > 3 cm (N = 19)**	20.2 ± 75.6		144 ± 268	
**No multifocal tumour (N = 22)**	1.6 ± 1.3	0.45	4.4 ± 4.6	0.41
**Multifocal tumour (N = 22)**	2.2 ± 3.4		5.9 ± 7.2	
**No lymphovascular invasion (N = 63)**	2.1 ± 3.1	0.6	5.5 ± 6.7	0.9
**Lymphovascular invasion (N = 5)**	1.4 ± 1.5		5.8 ± 4.9	

**Table 3:** Time to progression (TTP) and cancer-specific survival (CSS) mean values (± standard deviation) according to null or positive CTC count.

**CTCs**	**TTP (Months)**	***p*** **-Value**	**Events**	**Censured**	**Total**
0	136 ± 7.4	<0.001	14	55	69
≥1	26 ± 6.4		13	1	14
**CTCs**	**CSS (Months)**	***p*** **-Value**	**Events**	**Censured**	**Total**
0	148 ± 6.6	<0.001	6	62	64
≥1	61 ± 13		9	6	15

**Table 4:** Count of tdEVs in the patients studied.

tdEV Number	Patient Numbers	Percentage
0–6	58	69
7–14	14	17
≥15	12	14

**Table 5:** Time to progression (TTP) mean values (± standard deviation) according to CTC counts and tdEV threshold counts values.

CTCs	tdEVs	TTP (Months)	*p*-Value	Events	Censured	Total
0	<5	123 ± 5.9	0.25	5	32	37
	≥5	126 ± 11.8		8	23	31
≥1	<5	21.3 ± 5.5	0.71	6	0	6
	≥5	29.8 ± 16.9		8	1	9
0	<10	143.6 ± 6	0.06	9	49	58
	≥10	75 ± 19		4	6	10
≥1	<10	38 ± 8.8	0.001	8	1	9
	≥10	9 ± 1.3		6	0	6
0	<12	130 ± 6.9	0.56	11	50	61
	≥12	87 ± 22		2	5	7
≥1	<12	38 ± 8	0.001	8	1	9
	≥12	9 ± 1.4		6	0	6

**Table 6:** Cancer-specific survival (CSS) mean values (± standard deviation) according to CTCs counts and tdEVs threshold counts values.

CTCs	tdEVs	CSS (Months)	*p*-Value	Events	Censured	Total
0	<5	121 ± 8.6	0.38	33	4	37
	≥5	153 ± 7.8		29	2	31
≥1	<5	68 ± 7.5	0.48	3	3	6
	≥5	58 ± 18		6	3	9
0	<10	150 ± 6.5	0.80	5	53	58
	≥10	107 ± 16.2		1	9	10
≥1	<10	85 ± 17	0.01	4	5	9
	≥10	30.1 ± 9.6		5	1	6
0	<12	150 ± 6.4	0.68	5	56	61
	≥12	107 ± 16		1	6	7
≥1	<12	85 ± 17	0.01	4	5	9
	≥12	30.4 ± 9.7		5	1	6

**Table 7:** Time to first recurrence (TFR) mean values (± standard deviation) according to CTC and tdEV counts.

CTCs	tdEVs	TFR (Months)	*p*-Value
0	<10	89 ± 10	0.37
	≥10	33 ± 8	
≥1	<10	7.2 ± 1.8	0.48
	≥10	5 ± 0.6	

**Table 8:** Time to second recurrence (TSR) mean values according to CTC and tdEV counts.

CTC	tdEVs	TSR (Months)	*p*-Value
0	<10	110 ± 9.3	0.31
	≥10	43 ± 7.1	
≥1	<10	19 ± 3.8	0.29
	≥10	17 ± 3.3	

## Data Availability

The corresponding author will share data upon reasonable request.
